# Does Music Experience Impact the Vascular Endothelial Response to Singing?

**DOI:** 10.3390/brainsci15090996

**Published:** 2025-09-16

**Authors:** Mehri Bagherimohamadipour, Muhammad Hammad, Alexis Visotcky, Rodney Sparapani, Jacquelyn Kulinski

**Affiliations:** 1 Division of Cardiovascular Medicine, Medical College of Wisconsin, Milwaukee, WI 53226, USA; mbagherimo@mcw.edu (M.B.); mhammad@mcw.edu (M.H.); 2 CIBMTR^®^ (Center for International Blood and Marrow Transplant Research), Medical College of Wisconsin, Milwaukee, WI 53226, USA; avisotcky@mcw.edu; 3 Division of Biostatistics, Data Science Institute, Medical College of Wisconsin, Milwaukee, WI 53226, USA; rsparapa@mcw.edu

**Keywords:** singing, endothelial function, cortisol, elderly

## Abstract

**Background:** Vascular endothelial function is closely related to brain health, especially in individuals with cardiovascular risk factors. In a randomized, crossover clinical trial (NCT04121741), we have previously shown that 30 min of singing improves microvascular endothelial function in older adults with coronary artery disease. Here, we report on secondary and exploratory analyses, including (1) changes in cortisol and cytokine levels and their impact on vascular endothelial function, and (2) the impact of personal music experience on vascular function. **Methods:** Participants had three study visits separated by 2–7 days, according to a randomized, researcher-blinded, crossover, controlled design: (1) a 30-min period of live singing with an in-person music therapist, (2) a 30-min period of singing along to an instructional video and (3) a 30-min rest (control). Primary outcomes included macrovascular endothelial function assessed by brachial artery flow-mediated dilation (BA FMD%) and microvascular function assessed by peripheral arterial tonometry [Framingham reactive hyperemia index (fRHI) and reactive hyperemia index (RHI)]. Exploratory outcomes included (log) changes in salivary cortisol and cytokine (IL-6, TNF-α, IL-1β, IL-8) levels. Participants were asked to complete the Brief Music Experience Questionnaire (BMEQ), a 53-item validated self-report questionnaire designed to measure an individual’s overall experience with music. The BMEQ assesses how people perceive, react to, and engage with music in various aspects of their lives. **Results:** Sixty-five subjects (mean age 67.7 ± 6.6 years, 40% female) completed the study. Compared to those subjects completing the BMEQ (n = 31), there were no significant differences in age, sex, race, or presence of diabetes mellitus, hypertension, high cholesterol, heart failure, chronic kidney disease, or chronic respiratory disease in subjects who did not complete the BMEQ (n = 34). Total BMEQ score did not impact changes in BA FMD% (−3.49 ± 2.00, *p* = 0.086), changes in fRHI (0.58 ± 0.93, *p* = 0.535), or changes in RHI (0.73 ± 0.65, *p* = 0.262). When we decompose the sum of squares based on intervention, sex, race, and age, the BMEQ score does not predict changes in vascular function measures. In cross-over analyses, there were no acute changes in salivary cortisol or cytokine levels with 30 min of singing compared to control. Changes in IL-8 were directly related to changes in microvascular endothelial function (0.470 ± 0.184, *p* = 0.012 for RHI and 0.780 ± 0.248, *p* = 0.002 for fRHI). Changes in TNF-α were inversely related to changes in fRHI (−0.547 ± 0.263, *p* = 0.040). Changes in cortisol concentrations were not related to measures of vascular function. **Conclusions:** The beneficial changes in microvascular endothelial function are not modified by personal music experience in older subjects with known coronary artery disease. There were no changes in salivary cortisol or cytokine levels after 30 min of singing compared to control.

## 1. Introduction

Cardiovascular disease (CVD), which includes coronary heart disease, stroke, and peripheral arterial disease, is the leading cause of death (in most developed countries), threatening the health of the elderly [1]. A hallmark of aging is vascular disease driven by impairment of endothelial cell function. Endothelial cells form the interior of blood vessels, functioning both as a barrier and regulator to support vascular health. Vascular endothelial function is closely related to brain health, especially in individuals with cardiovascular risk factors. Endothelial dysfunction is implicated in the development and progression of various cerebrovascular diseases, including stroke and small vessel disease, and is a key feature in chronic conditions like atherosclerosis and hypertension [2]. Assessment of vascular endothelial function has proven to be a useful tool in translational research since vascular endothelial function strongly predicts adverse cardiovascular events in both primary and secondary prevention populations [3]. For instance, for every 1% increase in brachial artery flow-mediated dilation, there is an 8–13% lower risk of CVD events [4]. Furthermore, endothelial dysfunction (and CVD risk) is improved by interventions (i.e., healthy lifestyles, statin medications) known to reduce cardiovascular risk [5,6,7]. However, CVD in older adults is frequently complicated by age-related difficulties, including co-existing health conditions, frailty and deconditioning, disability, and other challenges, making involvement in physical exercise difficult. Alternative therapies to lower CVD burden and improve vascular and brain health outcomes are needed in this aging population.

Music as a therapeutic is attractive for a variety of reasons, including minimal risk to patients, ease of use, accessibility, and pervasiveness across culture. Most prior literature has examined the impact of music listening on health outcomes [8], including positive effects on mental health and psychological stress outcomes, as well as some favorable changes in basic vital signs such as heart rate, blood pressure, respiratory rate and heart rate variability [8,9]. Singing, a more active intervention (than passive music listening), may be more likely to influence physiological signals, such as the upregulation of oxytocin and endorphins, which improves mood and immune function [10]. Furthermore, the physiological demands of singing were found to be similar to walking at a moderately brisk pace [11], providing biological credibility that the health benefits of singing may intersect with that of exercise. However, unlike traditional physical exercise, the impact of singing on vascular health, especially in persons with known atherosclerotic vascular disease, has not been extensively studied.

In a randomized, crossover, controlled clinical trial, we have previously shown that 30 min of singing improves microvascular endothelial function in older adults with atherosclerotic coronary artery disease (CAD) [12]. The estimated improvement in microvascular endothelial function in the singing video arm of this clinical trial translates into a striking 25% reduction in cardiovascular risk [4,12]. The depth of one’s musical experience can potentially influence health outcomes, with active participation often leading to more profound effects on the immune system, for example, compared to passive listening [13]. For this reason, we set out to examine music experience as a modifier. Yet, traditional physical exercise benefits everyone’s cardiovascular health. Here, we report on secondary analyses to explore potential mechanisms for the health benefits of singing. We hypothesize that (1) immunomodulatory changes in cortisol and cytokines may explain the beneficial impact on vascular endothelial function and (2) one’s personal music experience may modify the impact of singing on vascular function.

## 2. Methods

### 2.1. General Study Design

Data was obtained from our previously completed clinical trial, Singing and Cardiovascular Health in Older Adults (NCT04121741) [12]. The detailed methodology describing this study has been outlined in the previously published paper [12]. In brief, sixty-five subjects, ages 55 to 79, with established atherosclerotic CAD participated in the study. Participants had the following three study visits, separated by at least 2 days, according to a randomized, investigator-blinded, crossover, control design: (1) a 30-min period of live singing with a music therapist, (2) a 30-min period of singing to an instructional video directed by a voice professor, and (3) a 30-min rest (control) period. Participants were seated for all three visits. Specific reporting guidelines for music-based interventions were followed to improve the transparency and specificity of reporting music-based interventions [14].

### 2.2. Singing (and Control) Interventions

A video series was specifically created and recorded for the purposes of this study. The video singing intervention included an instructional sing-along video displaying a voice professor playing the piano and directing an elderly student in singing. The 10-min vocal warm-up video included semi-occluded vocal tract (SOVT) activities, lip trills, humming, pitch modulation and face and tongue exercises. Full details of the video can be found in our prior publication [12]. The subjects selected 2 songs to sing for 10 min each from four different music genres including Folk (This Land is Your Land), Pop (Hey Jude), Country (Jolene), and Hymn (Amazing Grace)—varying in tempo, melodic contour, and rhythm to fill the full 10 minutes per song. Lyrics were displayed along the bottom of the video screen and laptop.

The live music intervention was an in-person singing session with a board-certified music therapist, who rotated between the keyboard or guitar depending on the subjects’ music selection. This session also included a 10-min vocal warm-up followed by two songs at 10 min each. Song selections were chosen by the subject from a multi-genre binder targeting older adults and including over 40 song options [12].

During the control intervention, subjects had a 30-min period of rest for which they were not allowed to sleep, watch television, read, browse their smartphone, or listen to music.

### 2.3. Measures of Vascular Endothelial Function

Vascular function measurements were performed before and after each study visit using the protocol previously described [12]. Macrovascular endothelial function was assessed using brachial artery flow-mediated dilation (FMD), a non-invasive method that evaluates changes in arterial diameter in response to reactive hyperemia induced by a short period of arterial occlusion [3]. FMD was measured by trained sonographers, who were blinded to the crossover treatment, using high-resolution ultrasonography with a 7.5–13 MHz probe. The results of FMD are expressed as the percentage change in post-stimulus arterial diameter compared to baseline (FMD%).

Microvascular endothelial function was assessed by digital peripheral arterial tonometry (PAT) using Endo-PAT 2000 device (Itamar Medical, Caesarea, Israel). PAT is a non-invasive technique that measures changes in pulse wave amplitude in response to reactive hyperemia [15]. It is expressed as the reactive hyperemia index (RHI) and Framingham reactive hyperemia index (fRHI) [16]. PAT and FMD measurements were performed simultaneously during each study visit.

All vascular function studies for the same study subject are performed by the same technician across all study visits. This minimizes inter-operator variability. In our laboratory, FMD measurements made twelve weeks apart show an average intra-observer variation of 1.1 ± 0.7% and inter-observer variation of 1.3 ± 1.1%. Intraclass correlation coefficients for intra- and inter-observer variation in measurements made 12 weeks apart range from 0.72 to 0.87. These reproducibility data are well-within accepted standards [17,18,19].

### 2.4. Salivary Cortisol and Cytokine Collection

To assess the effects of singing on objective measures of the stress response and the immune system, we measured salivary cortisol and cytokine levels including interleukin-6 (IL-6), tumor necrosis factor alpha (TNF-α), interleukin-1 beta (IL-1β), and interleukin-8 (IL-8) [20,21]. The subjects were fasted and asked to provide approximately half a teaspoon of saliva into a specialized collection tube, before and after each 30-min singing (and control) visit. All pre-intervention samples were collected between 7:00 and 8:00 a.m. All post-intervention samples were collected between 8:00 and 9:00 a.m. Samples were sent to an outside laboratory (Salimetrics SalivaLab) to have cortisol and cytokine levels measured. Salivary cortisol was measured using the Salimetrics Salivary Cortisol Assay Kit (Cat. No. 1-3002), and the cytokine panel was assayed using a proprietary electrochemiluminescence method developed and validated for saliva by Salimetrics. The average coefficient of variation in these measurements exceeded the applicable National Institute of Health guidance for Enhancing Reproducibility through Rigor and Transparency. A test volume of 25 µL was used for each determination, with two determinations performed per sample and the mean value reported. Salivary cortisol and cytokine concentrations were measured in µg/dL for cortisol and pg/mL for IL-1β, IL-6, IL-8, and TNF-α. To prevent outliers from unduly influencing the results, values exceeding 2.5 standard deviations from the cohort mean were removed from final analysis.

### 2.5. Borg Rate of Perceived Exertion (Borg RPE)

Borg RPE is a self-reported, user-friendly tool scaled from 6 (no exertion at all) to 20 (maximal exertion) [22]. Subjects’ effort and exertion were measured by the Borg RPE scale after each study visit to assess perceived level of exertion during singing (or control). The Borg RPE is the preferred method to measure intensity among those individuals who take medications (such as beta blockers or calcium channel blockers) that affect heart rate [23].

### 2.6. Brief Music Experience Questionnaire (BMEQ)

Participants were asked to complete the previously validated brief music experience questionnaire (BMEQ), a 53-item validated self-report questionnaire designed to measure an individual’s overall experience with music. The BMEQ assesses how people perceive, react to, and engage with music in various aspects of their lives. The questionnaire tests the following 6 aspects of music experience: (1) commitment to music (the centrality of pursuit of musical experiences in the person’s life); (2) innovative musical aptitude (self-reports of musical performance ability as well as the ability to generate musical themes and works); (3) social uplift (the experience of being uplifted in a group-oriented manner by music); (4) affective reactions (affective and spiritual reactions to music); (5) positive psychotropic effects (calming, energizing, integrating reactions); and (6) reactive musical behavior (motile reactions including humming and swaying along with music) [24]. Respondents’ choices on a five-point scale represent their level of agreement with survey statements from 1 (Very untrue) to 5 (Very true). Statements are intended to be relevant to non-musicians as well as musicians. Higher score on the BMEQ indicates a greater level of musical engagement and a more profound experience with music. This questionnaire was used with the permission of the authors.

The BMEQ was not part of the original study design, and we had to get an IRB amendment approved to update the informed consent for the questionnaire. Any subject who had already completed study participation was required to sign a consent addendum in order to complete the BMEQ. Some were contacted months to years after original study participation. Almost all participants completed the questionnaire via a REDCap email survey after study participation. Four participants completed the questionnaire during their last study visit.

### 2.7. Statistical Analysis

The Statistical Analysis System (SAS^®^ 9.4) was used for all statistical analyses. Crossover trials with three treatment arms have a widely accepted analysis plan that we followed [25]. First, with three treatment arms, the carry-over effect can be estimated from one time-point to the next (something that is not possible with two treatment arms). If the *p*-value for the carry-over effect is >0.1, then no carry-over is considered further. There are up to four components that are estimated with unbalanced analysis of covariance by regression (fixed effects only, no random effects) in the primary analysis in this order: (i) an intercept for each participant; (ii) the timing of the treatment (first, second or third); (iii) the treatment itself (singing with live music, singing along to instructional video, or control without singing); and (iv) the carry-over effect, if any. With this base model, we added a term for the BMEQ for a secondary model but, obviously, this was limited to those who completed the BMEQ.

Because FMD and RHI/fRHI were measured before and after each treatment, we analyzed the absolute change (after-before). Similarly, cortisol and cytokines were measured before and after each treatment. However, cortisol and cytokines are concentrations that can vary by orders of magnitude between subjects, so we took natural logarithms (base e) before differencing, i.e., log_diff = log(after) − log(before).

For FMD, RHI, fRHI, cortisol and cytokines, we identified outliers as >2.5 the study cohort’s standard deviation. Generally, the number of outliers removed were few for each outcome. Additionally, we estimated FMD, RHI, and fRHI regression coefficients for cortisol and cytokines with crossover methodology in two ways: (i) by removing outliers, and (ii) rank normalization without removing outliers. If the *p*-values for the treatment arms were relatively close between these two models, then we concluded that assumption of normally distributed errors in method (i) was accurate, and those results have been reported here. The significance level was adjusted to account for multiple comparisons (three measures of vascular function) using the Bonferroni correction, to minimize risk for type 1 error. The *p*-value for achieving statistical significance of our primary outcome was set at *p* < 0.016.

## 3. Results

Baseline characteristics are displayed in Table 1. Sixty-five subjects completed the study between 7 January 2020, and 18 August 2023. Please refer to the consort diagram in the original manuscript for full details on participant progression through the cross-over design [12]. The mean age of participants was 67.7 (±6.6) years with 40% female. Most of the participants were non-Hispanic (98.5%) and identified as White (87.5%). Physical limitation was ascertained by self-report with over 53% reporting some limitations. All participants had CAD, one of the mandatory inclusion criteria. Dyslipidemia (84.6%), hypertension (75.4%), and diabetes mellitus (29.2%) were common co-morbidities. Forty percent of subjects were prior tobacco smokers.

In crossover analyses, there were no acute changes in salivary cortisol or cytokine concentrations with 30 min of singing. Crossover estimates between the two different singing interventions and control are reported as absolute change in (post- to pre-intervention) salivary (log) cortisol and (log) cytokine concentrations, Table 2. For change (±SE) in cortisol, the live music intervention showed an estimated difference of 0.04 ± 0.06 (*p* = 0.471), and the video intervention showed an estimated difference of −0.06 ± 0.06 (*p* = 0.283) compared to control. For change in IL-1β, the live music intervention showed an estimated difference of −0.07 ± 0.10 (*p* = 0.482), and the video intervention showed an estimated difference of −0.04 ± 0.10 (*p* = 0.715) compared to control, and so on and so forth. There were no significant changes in salivary cortisol levels after 30 min of singing compared to control.

Thirty-one subjects completed the BMEQ, Table 1. There were no differences in age, sex, race, or presence of diabetes mellitus, hypertension, high cholesterol, heart failure, chronic kidney disease, or chronic respiratory disease between those subjects completing the BMEQ (n = 31) and those subjects not completing the BMEQ (n = 34). Total BMEQ score did not influence changes in BA FMD% (−3.49 ± 2.00, *p* = 0.086), changes in fRHI (0.58 ± 0.93, *p* = 0.535), or changes in RHI (0.73 ± 0.65, *p* = 0.262), Table 3. By analysis of covariance, we estimated the portion of total variability due to the BMEQ score and it was not associated with changes in BA FMD%, RHI, or fRHI (*p* of 0.016 used to indicate significance). For absolute change in Borg RPE, the live music intervention showed an estimated difference of 4.33 ± 0.28 (*p* < 0.0001), and the video intervention showed an estimated difference of 3.65 ± 0.28 (*p* < 0.00013) compared to control. Compared to the video intervention, the live music intervention led to higher BORG RPE scores (9.98 ± 0.28 and 10.66 ± 0.29, respectively) with a difference of 0.68 ± 0.28), *p* = 0.0167. The BORG RPE was not a mediator or moderator of vascular function.

The relationship between changes in cytokine concentrations and vascular function is displayed in Table 4. There is a direct relationship between change in IL-8 concentrations and change in fRHI, with an estimated difference of 0.78 ± 0.25 (*p* = 0.002). For TNF-α, there is a borderline significant inverse relationship with fRHI, with an estimated difference of –0.55 ± 0.26 (*p* = 0.040). For RHI, there was a significant direct relationship with IL-8 (0.47 ± 0.18, *p* = 0.012). The relationship between changes in cytokines and FMD were not significant (Supplementary Table S2a). The relationship between changes in cortisol levels and vascular function were not significant (Supplementary Tables S1 and S2b).

## 4. Discussion

In this secondary analysis of our clinical trial (NCT04121741), no measures of micro- or macro-vascular function were significantly related to music experience, suggesting that the physiological vascular benefits of singing may occur regardless of one’s personal engagement and/or experience with music. This is an encouraging finding and aligns with the key principle that singing is a physical activity. In other words, you do not have to be a professional athlete or singer to reap the cardiovascular benefits of exercise or singing activities. This finding increases the attractiveness of singing in health promotion as an accessible, low-cost, scalable, safe and largely enjoyable intervention for a wide range of older adults, irrespective of prior music experience. While very few studies explore differences in health outcomes between professional and amateur singers, numerous studies—particularly on community singing groups and interventions with older adults—demonstrate mental (improved well-being [10], decreased loneliness [26]) and physical (improved lung and cognitive [27] function) health benefits for participants across varying levels of singing ability [28,29]. Furthermore, a group singing format would likely be more cost-effective in terms of uptake and ease of implementation in outpatient cardiac rehabilitation settings. A pre-recorded virtual singing video series would also be practical for those who may have transportation issues and/or prefer to sing alone. This virtual format could be more practical to implement on a larger scale. Our multi-disciplinary team has future plans in place to investigate these additional formats and determine the optimal dose and frequency of these singing interventions.

In the present analysis, there were no significant changes in salivary cortisol levels after 30 min of singing compared to control. This may be related to the low intensity of the singing activities, as BORG RPE scores in the range achieved here (9.98 to 10.66) correspond to a low-intensity exercise. In general, moderate to high-intensity exercise causes a temporary increase in cortisol levels. However, the extent of the increase depends on the intensity, duration, and type of exercise [30]. The 30-min intervention may not be long enough to evoke measurable hormonal changes. Another possible explanation for the null cortisol finding could be that the parasympathetic activation with singing (i.e., through stimulation of the vagus nerve with vocal cord vibrations and innervation of the larynx and pharynx) [31] counterbalances the stress response of the singing (exercise) activity, helping to maintain cortisol levels and promote relaxation. In fact, a separate study in 32 older adults, mean age 64.3 years, singing just one song (mean singing duration 3 min and 50 s) resulted in a decrease in salivary cortisol levels [29]. Perhaps the short duration of the singing in this study (compared to 30 min in our singing interventions) was enough to stimulate the parasympathetic nervous system with little activation of the sympathetic nervous system, resulting in a reduction in cortisol levels.

There were no significant changes in salivary cytokine levels after 30 min of singing compared to control. This finding differs from a prior study of 193 subjects in populations affected by cancer (carers, bereaved carers, and patients) with mean age 56.9 years, showing increases in various cytokines (IL-2, IL-4, IFN-Ɣ, and TNF-α) after one hour of group singing in a choir format [32]. They also saw reductions in cortisol levels. It may be that the group singing format is more likely to modulate components of the immune system through improved mood, related to factors such as social cohesion and connectedness. Perhaps the longer duration of the intervention (1 h) was needed to see meaningful changes in the cytokine levels. The main limitation, however, of this positive study is the lack of a control arm or group for comparison, reducing the rigor of the study conclusions. We also considered that singing might provoke anxiety for some people. Anxiety is typically associated with unfavorable changes in pro-inflammatory cytokines [33] compared to healthy controls and therefore, may negate any potential improvements in these markers if anxiety were not a contributing factor. This is speculative, however, as we did not have any objective measurements of anxiety or emotion. Future research needs to consider the moderation or mediation of emotions (on health-related outcomes) interacting with singing. Singing in a group may lessen anxiety [34] associated with singing and would also be more scalable from a healthcare systems standpoint.

Immunomodulatory changes (in cortisol and cytokine levels) are not the sole mechanisms by which vascular endothelial function changes occur. We need to consider other complex biological processes involved, such as nitric oxide pathways and autonomic nervous system effects. Physical activity promotes increased nitric oxide (NO) secretion, which leads to vasodilation, enhancing microvascular blood flow perfusion, and regulating hemodynamics. The bioavailability of NO is determined by the balance between its enzymatic production and degradation by reactive oxygen species (ROS). Physical activity reduces activity of ROS-generating enzymes and augments endogenous antioxidant protection, thus decreasing levels of ROS, and enhancing NO bioavailability. NO works in synergy with a variety of hormones to regulate the microcirculation. For example, NO likely regulates glucose metabolism by stimulating insulin secretion and sensitivity, thereby improving microcirculatory vascular function [35]. Capillary proliferation is another factor that improves microcirculatory function. This process is regulated by the balanced control of pro-angiogenic and anti-angiogenic signals. Vascular endothelial growth factor (VEGF) is the most potent known positive regulator that increases capillary density by promoting division of microvascular endothelial cells, increasing angiogenesis, and enhancing microvascular permeability [36]. The mechanical stress from increased blood flow during physical activity (and singing?) enhances expression of VEGF mRNA. This could also be investigated in future studies of signing.

The autonomic nervous system (ANS) can also affect microvascular endothelial function by releasing vasoactive mediators that cause vasoconstriction (norepinephrine) or vasodilation (acetylcholine). Consequently, ANS imbalance could be a risk factor for cardiovascular disease, and sympathetic nervous activation represents a maladaptive phenomenon in vascular function and structural integrity [37]. Prior studies have reported a correlation between flow-mediated dilation and heart rate variability (HRV) in subjects with ischemic heart disease. The relationship, however, was not present in subjects with diabetes and subjects taking beta-blocking agents (which many patients with CAD take) [38]. If we extrapolate what we know about regular exercise, a longer singing intervention (weeks to months) could improve resting HRV due to greater ANS balance. Measuring these chronic alternations in HRV are likely more clinically meaningful (than acute changes) and should be considered in future studies of singing [12].

While there were no significant changes in salivary cytokine levels after 30 min of singing compared to control, physiologic exploratory analyses in our study did show a significant direct association between changes in IL-8 and microvascular function (both RHI and fRHI) whereby elevations in IL-8 correspond with improvements in microvascular endothelial function. IL-8 is known to play a complex role in vascular endothelial function, influencing both pro-angiogenic and even pro-inflammatory processes. It can promote endothelial cell migration, proliferation, and survival, and increase vascular permeability, all crucial aspects of angiogenesis (new blood vessel formation) but also potentially contributing to coronary plaque formation (pro-inflammatory) [39]. In this patient cohort with known CAD, angiogenesis is often part of the healing and recovery process. Angiogenesis leads to the development of collateral vessels in the heart muscle to improve blood flow to ischemic areas that are deprived of oxygen and nutrients. As part of this process, IL-8 stimulates endothelial cells to migrate and divide, which are essential steps in angiogenesis. Given our finding that IL-8 levels correspond to improvements in microvascular endothelial function in this study population, we may be able to speculate that angiogenesis (rather than pro-inflammation) is the predominant mechanism for this finding.

Additionally, salivary TNF-α was inversely associated with fRHI. This cytokine is typically considered pro-inflammatory and is released by immune cells, such as macrophages and T cells, in response to infection, injury or other inflammatory stimuli. In terms of vascular function, TNF-α mediated signaling initiates and accelerates atherogenesis, thrombosis, vascular remodeling, vascular inflammation, vascular oxidative stress and impaired nitro oxide bioavailability, all contributing to vascular dysfunction [40]. Therefore, our finding that lower levels of TNF-α are associated with improvements in microvascular endothelial function is substantiated. We do need to consider that circadian rhythms can affect cytokines levels despite morning testing. Morning samples are shaped by the preceding hours of sleep and stress hormones, rather than being a neutral baseline [41]. Our analyses, which include the ‘changes in’ cytokine levels, should mitigate this issue, at least partially.

To our knowledge, this is the first study to explore the interplay between singing, inflammatory markers, and vascular endothelial function in older adults with CAD. While several prior studies have reported cardiovascular benefits from music listening interventions [8,42], the specific act of singing—an active and engaging musical behavior—has received comparatively less attention. Our findings contribute to a growing body of literature that suggests singing can have physiological benefits, at least in part mediated by improvements in microvascular endothelial function. Changes in salivary cytokine levels did not mediate changes in microvascular function related to the singing interventions.

This study is not without limitations. As a secondary analysis of a clinical trial that was not powered for these mechanistic outcomes, the results should be interpreted with caution. The sample included only older adults, mostly white, with CAD, limiting generalizability to other populations. Furthermore, the study assessed only short-term effects; long-term vascular function outcomes of regular singing practice remain unknown. We also acknowledge the potential variability in cytokine responses, which may be influenced by factors such as time of day, recent physical activity, or acute stress levels [43]. However, our subjects completed all study visits with vascular function studies in the mornings and in the fasted state. Selection bias is possible—those who agree to sing may differ from those who do not. Finally, only 31 subjects completed the BMEQ. The reason for the low attrition for BMEQ is that we decided to collect this information after many of the subjects already completed study participation. In other words, the BMEQ was not part of the original study design. Any subject who had already completed study participation were required to sign a consent addendum in order to complete the BMEQ. Some were contacted months to years after original study participation. Given the small sample size completing the BMEQ, it is difficult to definitively draw conclusions from these results. But, if they are reliable, this supports the theoretic framework of “singing as exercise” whereby all people benefit from exercise (regardless of their like or dislike for it). We also acknowledge that the BMEQ may not capture relevant aspects of musical engagement for health outcomes. It is also possible that the sample cohort size of 64 subjects is underpowered for changes in salivary cytokine levels. Our cross-over design, where each subject has before and after measurements, is more advantageous with smaller sample sizes.

Despite these limitations, our findings offer preliminary evidence that a single 30-min singing session can benefit vascular endothelial function and that these effects are unrelated to personal music experience. This has potential clinical and community implications, as singing is a low-cost, culturally adaptable activity that may support cardiovascular health in aging populations. Future studies should aim to replicate these findings in larger, more diverse cohorts and assess the longitudinal and sustained effects of repeated singing sessions on vascular function. Additionally, research should explore the mechanistic pathways linking singing to vascular outcomes, including autonomic regulation, inflammatory signaling, and psychosocial factors such as mood and social connectedness.

## 5. Conclusions

The beneficial changes in microvascular endothelial function are not modified by personal music experience. In older subjects with known CAD, there were no changes in salivary cortisol or cytokine levels after 30 min of singing compared to control. Future studies need to explore the sustained vascular response to singing over weeks to months and consider the group singing format as a more tangible intervention in healthcare settings.

## Figures and Tables

**Table 1:** Baseline characteristics (*N* = 65).

	Total 65 (col%)	BMEQ 31 (col%)	No BMEQ 34 (col%)	*p*-Value
Age (years): mean, SD	67.7, 6.6	68.4, 6.7	66.8, 6.5	0.373
Female	26 (40.0)	16 (44.4)	10 (34.5)	0.415
Race				0.376
Black	7 (10.9)	3 (8.3)	4 (14.3)	
White	56 (87.5)	33 (91.7)	23 (82.1)	
Asian	1 (1.6)	0 (0.0)	1 (3.6)	
Unknown	1	0	1	
History of coronary artery disease				
Myocardial infarction	41 (63.1)	21 (58.3)	20 (69.0)	0.377
Coronary stent	47 (74.6)	21 (61.8)	26 (89.7)	0.011
Coronary artery bypass	18 (27.7)	12 (33.3)	6 (20.7)	0.258
Diabetes mellitus	19 (29.2)	9 (25.0)	10 (34.5)	0.403
Hypertension	49 (75.4)	28 (77.8)	21 (72.4)	0.618
High cholesterol	55 (84.6)	30 (83.3)	25 (86.2)	0.750
Chronic kidney disease	10 (15.4)	6 (16.7)	4 (13.8)	0.750
Chronic respiratory disease	18 (27.7)	12 (33.3)	6 (20.7)	0.258
Heart failure	12 (18.5)	6 (16.7)	6 (20.7)	0.678
Prior smoking	26 (40.0)	14 (38.9)	12 (41.4)	0.839
BMI: mean, SD	30.0, 8.3	30.1, 6.9	29.8, 10.0	
BMI category				0.987
Underweight < 18.5	4 (6.2)	2 (5.6)	2 (6.9)	
Healthy weight 18.5 < 25	12 (18.5)	7 (19.4)	5 (17.2)	
Overweight 25: <30	17 (26.2)	9 (25.0)	8 (27.6)	
Obese 30 or greater	32 (49.2)	18 (50.0)	14 (48.3)	
Physical or orthopedic limitations	35 (53.8)	20 (55.6)	15 (51.7)	0.758
Level of limitation				0.277
None/Minimal	51 (78.5)	29 (80.6)	22 (75.9)	
Somewhat	12 (18.5)	7 (19.4)	5 (17.2)	
Very	2 (3.1)	0 (0.0)	2 (6.9)	

All *p*-values > 0.05 are considered non-significant.

**Table 2:** Salivary cortisol/cytokines comparing singing interventions to control.

	Absolute (Post–Pre)		
	Estimate (SE)	95% CI	*p*-Value
Log cortisol			
Coach	−0.04 (0.06)	(−0.16, 0.08)	0.471
Video	−0.06 (0.06)	(−0.18, 0.06)	0.283
Log IL-1β			
Coach	−0.07 (0.10)	(−0.07, 0.10)	0.482
Video	−0.04 (0.10)	(−0.04, 0.10)	0.715
Log IL-6			
Coach	−0.17 (0.11)	(−0.40, 0.05)	0.121
Video	−0.13 (0.11)	(−0.35, 0.09)	0.230
Log IL-8			
Coach	−0.002 (0.10)	(−0.21, 0.20)	0.986
Video	0.01 (0.10)	(−0.19, 0.21)	0.928
Log TNF-α			
Coach	0.01 (0.10)	(−0.20, 0.22)	0.943
Video	0.06 (0.10)	(−0.15, 0.27)	0.571

Unbalanced ANOVA cross-over analysis adjusted for the order of intervention and carry-over. Units for cortisol concentrations µg/dL; units for cytokine concentrations pg/mL.

**Table 3:** Relationship between Brief Music Experience Questionnaire (BMEQ) score and vascular function.

	Absolute (Post–Pre)				
Parameter	Estimate	SE	95% CI	*t* Value	*p*-Value
BMEQ—total score					
fRHI	0.58	0.93	(−1.28, 2.44)	0.630	0.535
RHI	0.73	0.65	(−0.56, 2.30)	1.130	0.262
BA FMD %	−3.49	2.01	(−7.50, 0.52)	−1.740	0.086

Unbalanced ANOVA cross-over analysis adjusted for the order of intervention and carry-over.

**Table 4:** Relationship between changes in cytokine concentrations and vascular function.

	Absolute (Post–Pre)				
	Estimate	SE	95% CI	*t* Value	*p*-Value
**Framingham reactive hyperemia index (fRHI)**					
Log IL-1β	−0.19	0.26	(−0.70, 0.32)	−0.750	0.455
Log IL-6	0.13	0.14	(−0.16, 0.41)	0.880	0.381
Log IL-8	0.78	0.25	(0.28, 1.28)	3.150	0.002
Log TNF-α	−0.55	0.26	(−1.07, −0.02)	−2.080	0.040
**Reactive hyperemia index (RHI)**					
Log IL-1β	−0.20	0.19	(−0.57, 0.18)	−1.040	0.302
Log IL-6	0.15	0.11	(−0.06, 0.36)	1.440	0.155
Log IL-8	0.47	0.18	(−0.10, 0.84)	2.560	0.012
Log TNF-α	−0.26	0.19	(−0.65, 0.12)	−1.370	0.175

Unbalanced ANOVA cross-over analysis adjusted for the order of intervention and carry-over. Units for cytokine concentrations pg/mL.

## Data Availability

The raw data supporting the conclusions of this article will be made available by the authors on request.

## Supplementary Materials

The following supporting information can be downloaded at: https://www.mdpi.com/article/10.3390/brainsci15090996/s1, Table S1: Relationship between changes in cortisol concentrations and microvascular endothelial function; Table S2a: Relationship between changes in cytokine concentrations and macrovascular endothelial function; Table S2b: Relationship between changes in cortisol concentrations and macrovascular endothelial funcion.
