# Supplementary material for: Does Music Experience Impact the Vascular Endothelial Response to Singing?
Source: Brain Sci. 2025 Sep 16;15(9):996. doi: 10.3390/brainsci15090996 (PMC12468951; doi:10.3390/brainsci15090996)
Supplement: Supplementary file 1 [file brainsci-15-00996-s001.zip › brainsci-3823324-supplementary.docx]

SUPPLEMENTARY TABLES

| **Supplementary Table 1.  Relationship between changes in cortisol concentrations and microvascular endothelial function** | | | | | | |
| --- | --- | --- | --- | --- | --- | --- |
|  |  | **Absolute** (Post - Pre) | | | |  |
|  | | **Estimate** | **SE** | **t value** | **p-value** |  |
| **Framingham reactive hyperemia index (fRHI)** | |  |  |  |  |  |
| **Log cortisol** |  | -0.03 | 0.27 | -0.110 | 0.914 |  |
|  |  | **Absolute** (Post - Pre) | | | |  |
|  |  | **Estimate** | **SE** | **t value** | **p-value** |  |
| **Reactive hyperemia index (RHI)** | |  |  |  |  |  |
| **Log cortisol** |  | 0.09 | 0.20 | 0.460 | 0.648 |  |
| Unbalanced ANOVA cross-over analysis adjusted for the order of intervention and carry-over.  Units for cortisol concentrations µg/dL. | | | | | | |

| **Supplementary Table 2a.  Relationship between changes in cytokine concentrations and macrovascular endothelial function** | | | | | | |
| --- | --- | --- | --- | --- | --- | --- |
|  |  | **Absolute** (Post - Pre) | | | |  |
|  | | **Estimate** | **SE** | **t value** | **p-value** |  |
| **Brachial artery flow-mediated dilation (FMD)** | |  |  |  |  |  |
| **Log IL-1**β |  | -0.26 | 0.64 | -0.400 | 0.687 |  |
| **Log IL-6** |  | 0.02 | 0.37 | 0.050 | 0.958 |  |
| **Log IL-8** |  | -0.55 | 0.63 | -0.870 | 0.388 |  |
| **Log TNF-α** |  | 0.55 | 0.71 | 0.780 | 0.439 |  |
| Unbalanced ANOVA cross-over analysis adjusted for the order of intervention and carry-over.  Units for cytokine concentrations pg/mL. | | | | | | |

| **Supplementary Table 2b.  Relationship between changes in cortisol concentrations and macrovascular endothelial function** | | | | | | |
| --- | --- | --- | --- | --- | --- | --- |
|  |  | **Absolute** (Post - Pre) | | | |  |
|  | | **Estimate** | **SE** | **t value** | **p-value** |  |
| **Brachial artery flow-mediated dilation (FMD)** | |  |  |  |  |  |
| **Singing intervention** |  |  |  |  |  |  |
| Coach |  | -0.11 | 0.43 | -0.250 | 0.802 |  |
| Video |  | -0.13 | 0.43 | -0.290 | 0.773 |  |
| **Log cortisol** |  | 0.14 | 0.65 | 0.220 | 0.826 |  |
| Unbalanced ANOVA cross-over analysis adjusted for the order of intervention and carry-over.  Units for cortisol concentrations µg/dL. | | | | | | |
